# Generation and Evaluation of Novel Biomaterials Based on Decellularized Sturgeon Cartilage for Use in Tissue Engineering

**DOI:** 10.3390/biomedicines9070775

**Published:** 2021-07-04

**Authors:** Olimpia Ortiz-Arrabal, Ramón Carmona, Óscar-Darío García-García, Jesús Chato-Astrain, David Sánchez-Porras, Alberto Domezain, Roke-Iñaki Oruezabal, Víctor Carriel, Antonio Campos, Miguel Alaminos

**Affiliations:** 1 Department of Histology and Tissue Engineering Group, Faculty of Medicine, University of Granada and Instituto de Investigación Biosanitaria ibs.GRANADA, E18016 Granada, Spain; olimpiaortiz@correo.ugr.es (O.O.-A.); garciagarciaoscar2b@gmail.com (Ó.-D.G.-G.); jchato@ugr.es (J.C.-A.); david.s.p.94@gmail.com (D.S.-P.); 2 Doctoral Programme in Biochemistry and Molecular Biology, University of Granada, E18071 Granada, Spain; 3 Department of Cell Biology, Faculty of Sciences, University of Granada, E18071 Granada, Spain; rcarmona@ugr.es; 4 Department of I+D, Caviar de Riofrío, Riofrío, E18313 Granada, Spain; alberto@caviarderiofrio.com; 5 Andalusian Network for the Design and Translation of Advanced Therapies (ANd&tAT), E41092 Seville, Spain; roke.oruezabal@gmail.com

**Keywords:** cartilage, tissue engineering, sturgeon, decellularization

## Abstract

Because cartilage has limited regenerative capability, a fully efficient advanced therapy medicinal product is needed to treat severe cartilage damage. We evaluated a novel biomaterial obtained by decellularizing sturgeon chondral endoskeleton tissue for use in cartilage tissue engineering. In silico analysis suggested high homology between human and sturgeon collagen proteins, and ultra-performance liquid chromatography confirmed that both types of cartilage consisted mainly of the same amino acids. Decellularized sturgeon cartilage was recellularized with human chondrocytes and four types of human mesenchymal stem cells (MSC) and their suitability for generating a cartilage substitute was assessed ex vivo and in vivo. The results supported the biocompatibility of the novel scaffold, as well as its ability to sustain cell adhesion, proliferation and differentiation. In vivo assays showed that the MSC cells in grafted cartilage disks were biosynthetically active and able to remodel the extracellular matrix of cartilage substitutes, with the production of type II collagen and other relevant components, especially when adipose tissue MSC were used. In addition, these cartilage substitutes triggered a pro-regenerative reaction mediated by CD206-positive M2 macrophages. These preliminary results warrant further research to characterize in greater detail the potential clinical translation of these novel cartilage substitutes.

## 1. Introduction

The generation of human tissue-like substitutes by tissue engineering (TE) is based on the combination of human cells with biocompatible biomaterials acting as scaffolds [1,2,3]. Ideally, biomaterials used in TE should be biomimetic, i.e., able to mimic the structure and biological properties of the native extracellular matrix (ECM), support cell attachment, and promote tissue regeneration [4]. Among the multiple biomaterials used in TE, natural scaffolds have several advantages, including their availability, high biocompatibility, presence of cell-binding motifs and bioactive cues, histoarchitecture, and potential recognition and metabolic processing by the body [4].

Different biomaterials have been used to generate bioartificial substitutes of human cartilage as advanced therapy medicinal products for the treatment of severe cartilage defects [5,6]. Human cartilage consists of a specialized cell population of chondrocytes immersed within a dense ECM containing abundant collagen fibers and proteoglycans. This structure is commonly damaged due to trauma, age-related degeneration or autoimmune diseases, among other causes, and osteochondral lesions are common clinical problems for orthopedic surgeons worldwide [7]. Although TE offers a promising therapeutic alternative for these patients, a perfect substitute for human cartilage has not been generated in the laboratory. In fact, ex vivo reproduction of the fine structure of native cartilage is difficult, and most biomaterials used to date are unable to support the large mechanical forces and weights borne by native cartilage [8]. In addition, most hydrogels used in cartilage TE show rapid degradation, which may hinder their in vivo use [9]. For these reasons, the search for a fully functional biomaterial is currently an active area of research in cartilage TE.

Biomaterials obtained by decellularizing natural products have been widely used in TE. Decellularized materials able to preserve the native ECM composition offer the advantage of being highly biomimetic to native tissue, which favors cell growth and viability as well as tissue repair and remodeling [10,11]. Interestingly, most structural and functional proteins of the ECM are known to be highly conserved among species—a characteristic that facilitates their implantation in a different organism with a very low risk of immunological rejection [12]. Various decellularized tissues have been investigated for cartilage TE, such as adipose tissue [13], umbilical cord [14], and hyaline cartilage [15].

Another possible source of cartilage for use in TE is the sturgeon chondral endoskeleton. The endoskeleton of this ancestral fish (order *Acipenseriformes*) is composed mostly of cartilage, making it a good source of tissue for research [16,17]. Previous work has identified some of the molecules in sturgeon ECM, including collagen [18,19] and chondroitin sulfate [20], which have been isolated and characterized. Although very little information is available on the general structure and composition of sturgeon cartilage, this material has been recently used in TE with promising results [21,22].

A notable limitation in human cartilage TE is the difficulty of obtaining an adequate source of viable cells and culturing human chondrocytes ex vivo [23]. In contrast, adult mesenchymal stem cells (MSC) can be isolated easily and efficiently from several sources, and their intrinsic proliferation and differentiation potential into several cell lineages makes them excellent candidates for the generation of bioartificial tissues by TE with an alternative cell source [24]. The chondrogenic potential of human MSC has been demonstrated [25], and this property opens the door to the use of MSC to generate tissue-like cartilage substitutes by TE.

The aim of this study was to investigate the potential usefulness of sturgeon cartilage as a scaffold for human cartilage TE. For this purpose, we first obtained a decellularized sturgeon cartilage scaffold. Then this scaffold was recellularized with human chondrocytes and with four different types of human MSC, and ex vivo and in vivo characterization studies were done to determine the potential usefulness of these novel tissue-like cartilage substitutes for TE.

## 2. Materials and Methods

### 2.1. Human and Sturgeon Collagen Sequence Alignment

To determine the homology between human and sturgeon collagen, we obtained their protein sequences from the National Center for Biotechnology Information (NCBI) database. For sturgeon collagen, the only sequences available in the database were *Acipenser baerii* and *Acipenser schrenckii* alpha-1 and alpha-2 chains of type I collagen, and *Acipenser naccarii* alpha-1 chain of type II collagen. After the corresponding human sequences were obtained, human and sturgeon sequences were aligned with MultAlin software [26], and the level of homology was calculated for each type of sequence with MAFFT (multiple alignment using fast Fourier transform) developed by EMBL-EBI.

### 2.2. Biochemical Characterization of Human and Sturgeon Cartilage with Ultra-Performance Liquid Chromatography

Sturgeon cartilage used in the present study was obtained from the head region of adult *Acipenser naccarii* slaughtered for human consumption (average age was 5-year-old and average size was 1.5 m). The heads of fishes were provided by the Caviar de Riofrío fish farm located in Riofrío, Granada, Spain. When the samples arrived at the laboratory, the skin was removed and the head chondral skeleton was obtained and cleaned with sterile materials and reagents, and then kept frozen at −20 °C. The main amino acid composition of sturgeon cartilage was determined by ultra-performance liquid chromatography (UPLC) with a Waters Acquity UPLC™ H-Class system (Waters, Manchester, UK) coupled to a SYNAPT G2 HDMS Q-TOF high-resolution system. Tissue samples were lyophilized, and equivalent amounts of each sample type (sturgeon cartilage and control human cartilage) were hydrolyzed for 30 s in 5 mL 6N HCl in a nitrogen atmosphere. The mixture was then incubated at 122 °C for 22 h and filtered, and 10 µL of this solution was mixed with 70 µL buffer and 20 µL derivatization agent (to increase volatility). One microliter of this solution was injected in the column and analyzed to acquire the different spectra. A standard solution containing 17 amino acids was also analyzed as a reference.

### 2.3. Obtaining Novel Biomaterials by Sturgeon Cartilage Decellularization

For decellularization, the sturgeon head cartilage was thawed and disks 8 mm in diameter and approximately 1 mm thick were obtained with a biopsy punch (Kay Medical, Seki, Japan) and a no. 10 surgical blade. These disks were immersed for 10 min in a solution consisting of 10 mL phosphate buffered saline (PBS) (Merck/Sigma-Aldrich, Darmstadt, Germany) with antibiotics/antimycotics to prevent contamination (Merck/Sigma-Aldrich). Some of these disks (n = 7) were stored as native controls of sturgeon cartilage (S-CTR). The rest of the disks (n = 33) were subjected to decellularization.

In order to decellularize the sturgeon cartilage disks, we used a protocol based on a combination of several previously described protocols [27,28,29]. First, disks were incubated in double-distilled water (ddH_2_O) at room temperature for 24 h with slight agitation to induce osmotic cell lysis [27]. Then the disks were transferred to 1% sodium-dodecyl-sulfate (SDS) solution (Thermo Scientific, Waltham, MA, USA) to remove the cytoplasmic and nuclear material. To eliminate the SDS, samples were washed 3 times (30 min each) in ddH_2_O at 4 °C. Then they were transferred to 3% Triton X-100 solution (Merck/Sigma-Aldrich) [29] to promote lipid dissociation. After three washes, the disks were incubated in 4% sodium deoxycholate (SDC) solution (Merck/Sigma-Aldrich) to dissociate intracellular proteins, and washed three times. Lastly, enzymatic treatment was applied twice with 100 mg/L DNase (Merck/Sigma-Aldrich) and 20 mg/L RNase (Merck/Sigma-Aldrich) [29] in PBS at pH 7.5, 37 °C, for 45 min. The reaction was stopped with cold PBS, and the samples were washed 5 times in PBS for 15 min to eliminate any enzyme residues. All detergents were diluted in ddH_2_O to avoid water loss during the process, which might have affected the tissue structure. All incubations with detergents lasted for 24 h and were done at room temperature. Decellularized cartilage disks (DCD) were stored in PBS at 4 °C until use.

To assess the efficiency of the decellularization process, DNA was extracted in triplicate from the decellularized materials with a QIAamp DNA Mini Kit (Qiagen, Hilden, Germany), according to the manufacturer’s instructions, and was then quantified by measuring absorbance at 260/280 nm with a NanoDrop 2000 spectrophotometer (Thermo Scientific). To determine the presence of cell nuclei and DNA remnants, histological sections were obtained as described below, and samples were stained with 4′,6-diamidino-2-phenylindole (DAPI) (Vector Laboratories, Burlingame, CA, USA). Histological and histochemical analyses were used to determine the preservation of ECM molecules after decellularization (see below).

### 2.4. Cell Cultures and Recellularization

Primary cultures of 4 different types of human MSC and human chondrocytes were obtained from human biopsies according to standard enzymatic digestion protocols as previously described [30]. MSC obtained from adipose tissue (ADSC), bone marrow (BMSC) and dental pulp (DPSC) were cultured in Dubelcco’s Modified Eagle’s Medium (DMEM) supplemented with 10% fetal bovine serum (FBS) and 1% antibiotics/antimycotics (all from Merck/Sigma-Aldrich). Umbilical cord Wharton Jelly MSC (WJSC) were cultured in AmnioMAX™ medium (Thermo Scientific) as previously reported [31]. Human chondrocytes were isolated from three small biopsies taken from the hip joint of a 65-year-old patient subjected to hip replacement due to hip fracture. Biopsies were subjected to collagenase enzymatic digestion as previously described [32], and primary cell cultures were cultured with expansion medium consisting of a 1:1 mixture of DMEM and F-12 HAM media supplemented with 10% FBS, 2 mM L-glutamine and 1% antibiotics/antimycotics (all from Merck/Sigma-Aldrich).

The cultures were incubated at 37 °C with 5% CO_2_, and the medium was changed every 3 days. Cells were dissociated before reaching confluence with 0.05% trypsin-ethylenediaminetetraacetic solution (Thermo Scientific). All MSC cultures were characterized according to the criteria of the International Society for Cellular Therapy, and we confirmed the positive expression of the surface markers CD90, CD105 and CD73, the negative expression of CD45 and the multilineage differentiation potential, including the chondrogenic differentiation of each MSC type.

To generate recellularized cartilage disks (RCD), 30,000 cells of each type were subcultured on the surface of each DCD (n = 7 for each cell type). To facilitate the recellularization process, DCD were placed on 3.5% type I agarose molds 8 mm in diameter to prevent the disks from moving during the cell attachment process. The RCD were incubated for 2–3 h at 37 °C with 5% CO_2_ to promote cell adhesion. Then 1 mL of each specific cell culture medium was added to each well, and the RCD were incubated for 24 h. The RCD recellularized with each cell type were designated RCD-ADSC, RCD-BMSC, RCD-DPSC, RCD-WJSC or RCD-HC according to the cell type used for recellularization. All RCD were maintained in ex vivo culture for up to 4 weeks with standard cell culture protocols using the same culture media that are described above for cell expansion.

### 2.5. In Vivo Evaluation

To determine the in vivo biocompatibility of each cartilage substitute, DCD and RCD were grafted in laboratory animals (n = 18). Each disk was cut in half prior to surgery and grafted in 6-week-old male immune-deficient athymic Nude-*Foxn1nu* mice. Briefly, the animals were deeply anesthetized with ketamine and acepromazine, and the different cartilage substitutes were grafted subcutaneously in the dorsal area of each animal. Each mouse received one graft of one sample of each type (DCD, RCD-ADSC, RCD-BMSC, RCD-DPSC, RCD-WJSC or RCD-HC). The animals were observed for 60 days, and the presence of any side effects (necrosis, infection, hemorrhage, tumorigenesis, rejection, etc.) was assessed daily in each animal. After the follow-up period, the animals were euthanatized and the grafted implants and surrounding tissues were harvested for histological analysis.

### 2.6. Histology and Histochemistry

All sturgeon cartilage samples (S-CTR, DCD and RCD) maintained ex vivo and in vivo were fixed in 4% formaldehyde for histological analysis. Human native epiglottis was used as a control for normal human cartilage (H-CTR). The samples were dehydrated in increasing concentrations of ethanol (70–100%), cleared in xylol, and embedded in paraffin, then 5-µm tissue sections were obtained with a microtome. The tissue sections were rehydrated and stained with hematoxylin-eosin (H-E) to assess the general structure in different samples, or with DAPI to identify cell nuclei. For scanning electron microscopy (SEM), DCD were fixed in 2.5% glutaraldehyde, dried using the critical point method, gold sputter-coated and analyzed with a Quanta 200 microscope (FEI, Eindhoven, The Netherlands).

To evaluate the presence of specific ECM components in tissues, several histochemical methods were used in accordance with previously published protocols [33,34,35]. Briefly, cartilage proteoglycans were identified by toluidine blue (TB) and alcian blue (AB) histochemistry, mature collagen fibers were stained with picrosirius red (PR), and elastic fibers were detected by orcein (OR) histochemistry. After staining, images were obtained with an Eclipse 90i microscope (Nikon, Tokyo, Japan) under the same conditions (magnification, exposure time, contrast, etc.) for all samples prepared with the same histochemical method.

### 2.7. Immunohistochemistry

Tissue sections were deparaffinized and rehydrated before the immunohistochemical analyses. For antigen retrieval, sections were treated with 20% Triton X-100 in ddH_2_O and proteinase K (Interchim™, Montluçon, France) for 30 min. Endogenous peroxidase was quenched with H_2_O_2_.

For type II collagen, KI-67, Caspase 7, CD4, CD8 and CD206 detection, unspecific sites were first blocked with 1× casein and horse serum for 15 min at room temperature. Then the sections were incubated overnight at 4 °C with primary antibodies against collagen II (Merck-Millipore; AB2036, 1:250 dilution, Burlington, MA, USA), KI-67 (Master Diagnostica, Granada, Spain, ref. MAD-00310QD, prediluted), Caspase 7 (Abcam, ref. ab69540, 1:100 dilution), CD4 (Abcam, Cambridge, UK, ref. ab237722, 1:4000 dilution), CD8 (Abcam, ref. ab237709, 1 µg/mL dilution), or CD206 (Abcam, ref. ab64693, 1:800 dilution). Negative controls were treated with PBS instead of the primary antibody. In all cases, sections were then incubated with a ready-to-use horse anti-rabbit antibody labelled with peroxidase (Vector Laboratories; MP-7401) for 1 h. The Diaminobenzidine (DAB) Substrate Kit (Vector Laboratories; SK-4100) was used for 40 s to detect antibody binding. The reaction was stopped with ddH_2_O. Lastly, the samples were counterstained with Harry’s hematoxylin for 15 s followed by washing for min in tap water. After staining, images were obtained with an Eclipse 90i microscope (Nikon) under the same conditions (magnification, exposure time, contrast, etc.) for all samples prepared with the same histochemical method.

### 2.8. Quantification and Statistical Analysis

The ECM components were quantified in samples processed for histochemical and type II collagen immunohistochemical assays with ImageJ software (National Institutes of Health, USA) as previously described [34,35,36]. Briefly, we selected 10 random points in each image with the software’s multi-point tool, and average intensity was then calculated automatically. Means and standard deviations were calculated for each component and each type of sample. For KI-67, CD4, CD8 and CD206 immunohistochemistry, the number of cells showing positive and negative staining signals was determined in histological images. In brief, an average of 100 cells were counted in each sample, and the number of cells showing positive and negative staining signal for each immunohistochemical method were determined. Then, the percentage of positive cells was calculated as the number of positive cells divided by the total number of cells, multiplied by 100.

To compare the quantitative results obtained for each sample type with control S-CTR and non-grafted DCD, we used ANOVA with Tukey’s range test. *p* values below 0.05 were considered statistically significant, and all tests were two-tailed. The comparisons were done with Real Statistics Resource Pack software (Release 7.2), available at www.real-statistics.com.

## 3. Results

### 3.1. Comparative Analysis of Human and Sturgeon Cartilage Proteins

To determine the homology between human and sturgeon cartilage at the molecular level, we first compared previously known sequences for type I and II collagen from *Homo sapiens* and *Acipenser* sp. This in silico sequence analysis showed that the human sequence for the alpha-1 chain of type I collagen had 84.24% homology with the sturgeon sequence at the protein level. For the alpha-2 chain, we found 65.29% homology between the two species, and the alpha-1 chain of type II collagen showed 81.46% homology. The protein alignments are shown in Supplementary Materials Figure S1.

**Figure 1:**
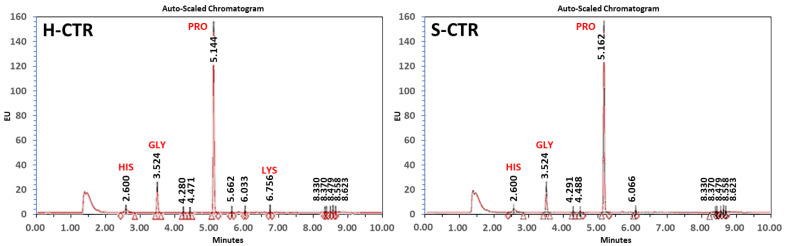
Ultra-performance liquid chromatography analysis of human native cartilage (H-CTR) and sturgeon native cartilage (S-CTR). The main peaks correspond to histidine (HIS), glycine (GLY), proline (PRO) and lysine (LYS). The numbers correspond to retention times (RT) obtained for each peak.

Analysis of the amino acid content of both types of cartilage with UPLC showed that sturgeon cartilage was very similar in composition to human cartilage. As shown in Figure 1, the predominant amino acid in both species was proline, followed by glycine and histidine, with human cartilage containing small amounts of lysine. Other amino acids were found at very low or undetectable levels.

### 3.2. Characterization of Native Human and Sturgeon Control Cartilage

**Figure 2:**
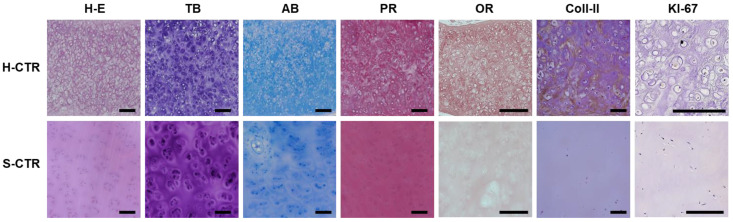
Histological analysis of control H-CTR and S-CTR with hematoxylin-eosin staining (H-E), toluidine blue (TB), alcian blue (AB), picrosirius red (PR) and orcein (OR) histochemistry, and immunohistochemistry for type II collagen (Coll-II) and KI-67 proteins. Scale bars: 200 µm.

The general structure of sturgeon cartilage was characterized by H-E, histochemistry and immunohistochemistry, and compared to human cartilage. As shown in Figure 2, cartilage in both species consisted of a dense ECM containing an abundant cell population that formed isogenic groups. Cartilage cells appeared to be more abundant in human cartilage, but were smaller compared to sturgeon cartilage, in which cell density was lower. When we analyzed the ECM by TB and AB histochemistry, we found that both H-CTR and S-CTR contained abundant proteoglycans. However, the proteoglycans were distributed homogeneously in H-CTR, with higher concentrations near the isogenic groups and lower concentrations in the inter-isogenic areas. In contrast, proteoglycans in S-CTR showed a more heterogeneous distribution in the inter-isogenic areas and were less abundant near the isogenic groups. We also found that collagen fibers identified by PR histochemistry were abundant in both species, with a more regular and homogeneous distribution in S-CTR compared to H-CTR. Lastly, OR revealed that H-CTR contained abundant elastic fibers in the ECM, whereas S-CTR was devoid of these fibers. After the control cartilage was analyzed histochemically, we carried out immunohistochemical analyses with specific primary antibodies. The results showed low amounts of type II collagen in H-CTR and very low amounts in S-CTR. In addition, we found that all chondrocytes in H-CTR and S-CTR were negative for the cell proliferation marker KI-67.

### 3.3. Obtaining Novel Biomaterials by Sturgeon Cartilage Decellularization

**Figure 3:**
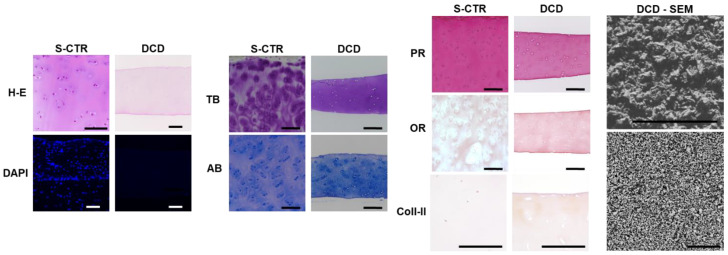
Histological analysis of S-CTR and decellularized cartilage disks (DCD) with H-E, DAPI, histochemistry for TB, AB, PR and OR, and immunohistochemistry for Col-II. DCD - SEM: DCD analyzed with scanning electron microscopy at two different magnifications. Scale bars: 200 µm.

Application of the decellularization protocol described here allowed us to obtain novel decellularized biomaterials. Decellularization efficiency was evaluated by quantifying the residual DNA in DCD (19.92 ± 5.79 ng/mg dry weight of tissue), which is consistent with the requirements of decellularized tissues [37]. These results were confirmed by H-E and DAPI staining of DCD, which showed that the decellularization process was able to eliminate all nuclei from the tissue compared to S-CTR (Figure 3), and demonstrated that the general structure of sturgeon cartilage was preserved after decellularization. In addition, our SEM analyses showed that the surface of the DCD was porous.

**Figure 4:**
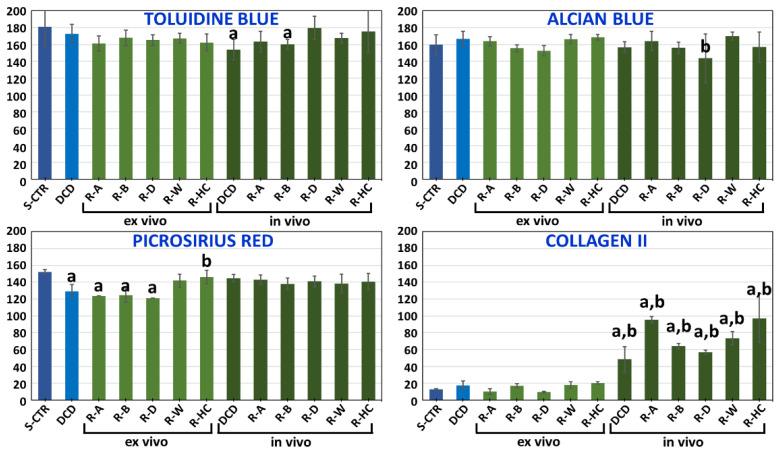
Quantitative analysis of proteoglycans as determined by toluidine blue and alcian blue, and of collagen fibers as determined by picrosirius red and type II-collagen, in S-CTR, DCD and RCD maintained ex vivo and grafted in vivo. Values correspond to reaction intensity for each histochemical or immunohistochemical method. R-A: RCD recellularized with MSC obtained from adipose tissue (ADSC); R-B: RCD recellularized with bone marrow MSC (BMSC); R-D: RCD recellularized with dental pulp MSC (DPSC); R-W: RCD recellularized with umbilical cord Wharton jelly MSC (WJSC); R-HC: RCD recellularized with human chondrocytes. a: Significantly different from S-CTR; b: Significantly different from DCD.

When specific ECM components were analyzed in DCD, we found that the decellularization process maintained most ECM components in the native structure. As seen in Figure 3 and Figure 4, decellularized cartilage showed intense TB and AB staining, which indicated a high concentration of proteoglycans, and the differences compared to native tissue were not statistically significant. However, detection of collagen fibers with PR revealed a significantly lower collagen content in DCD compared to S-CTR tissue. Our analysis of elastic fibers with OR showed that both the S-CTR and DCD were devoid of this type of fiber. Lastly, the low amounts of type II collagen found in S-CTR were also found in DCD, with nonsignificant differences between these two types of sample.

### 3.4. Ex Vivo Characterization of Recellularized Cartilage Disks

**Figure 5:**
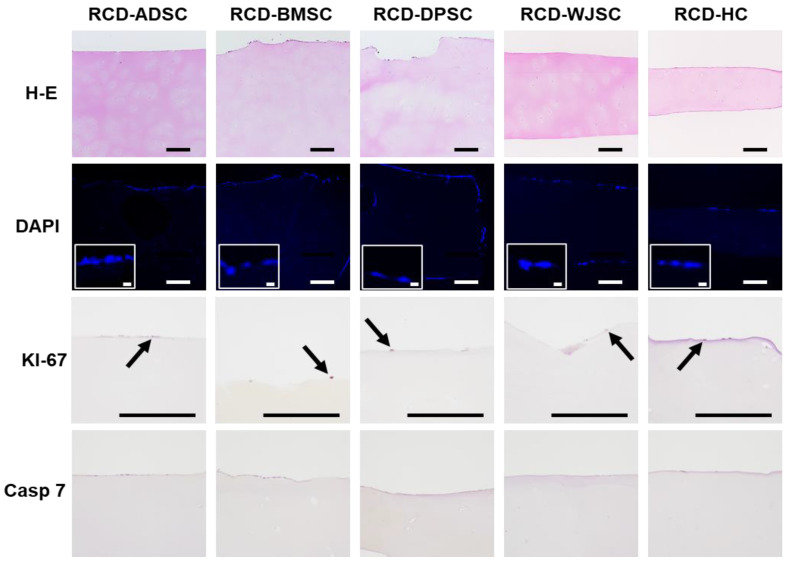
Histological analysis of recellularized cartilage disks (RCD) maintained in ex vivo culture, with H-E, DAPI and immunohistochemistry for KI-67 and Caspase 7 (Casp 7). Representative cells with positive staining for DAPI are shown in higher magnification inserts, and some KI-67-positive cells are highlighted with arrows. RCD-ADSC: RCD recellularized with adipose tissue MSC; RCD-BMSC: RCD recellularized with bone marrow MSC; RCD-DPSC: RCD recellularized with dental pulp MSC; RCD-WJSC: RCD recellularized with umbilical cord Wharton Jelly MSC; RCD-HC: RCD recellularized with human chondrocytes. Scale bars: 200 µm (20 µm for the high-magnification DAPI inserts).

After recellularization, all RCD showed a single layer of cells on the disk surface with positive DAPI staining (Figure 5). Interestingly, analysis of the cell proliferation marker KI-67 disclosed positive signals in only a few cells (less than 1%) regardless of the type of human MSC used for recellularization, and all cells were negative for the apoptosis marker Caspase 7.

**Figure 6:**
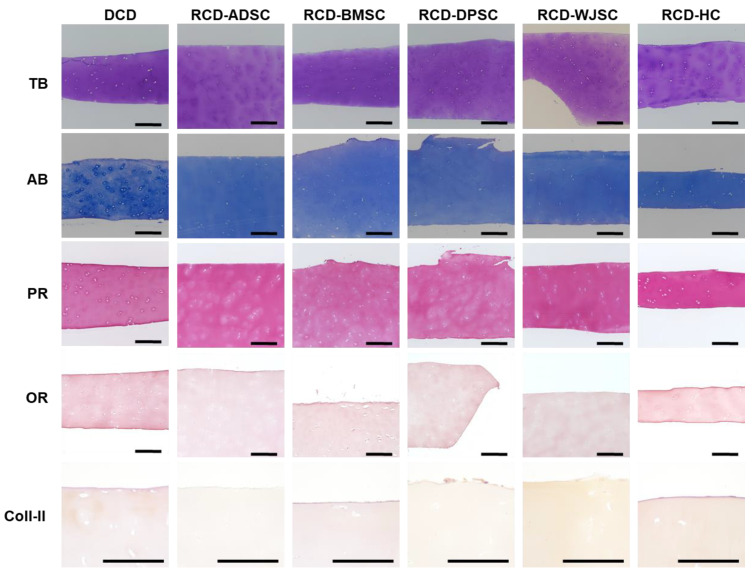
Histochemical and immunohistochemical analysis of the extracellular matrix in RCD maintained in ex vivo culture using TB, AB, PR and OR histochemistry and Coll-II immunohistochemistry. DCD are shown again at the left side for comparative purposes. Scale bars: 200 µm.

When the main ECM components were analyzed in RCD maintained ex vivo (Figure 4 and Figure 6), we observed very few differences between DCD and RCD. First, we found that proteoglycan content determined by TB and AB remained constant after recellularization, and the differences compared to DCD and S-CTR were not statistically significant. Quantification of mature collagen fibers stained with PR showed that the content of these fibers was similar in DCD and RCD-ADSC, RCD-BMSC, and RCD-DPSC. In all these samples the content was significantly lower than in S-CTR. However, a higher collagen content was found in RCD-WJSC, which was similar to S-CTR, and especially in RCD-HC, in which the content was significantly higher than in DCD and similar to S-CTR. Our analysis of elastic fibers with OR showed that all RCD were devoid of these fibers after 4 weeks of follow-up, as was the case for DCD and S-CTR. Lastly, our quantitative analysis of type II collagen showed that the low amount of this component seen in S-CTR and DCD remained present in all RCD samples maintained ex vivo, with nonsignificant differences among these samples.

### 3.5. In Vivo Characterization of Decellularized Cartilage Disks and Recellularized Cartilage Disks

**Figure 7:**
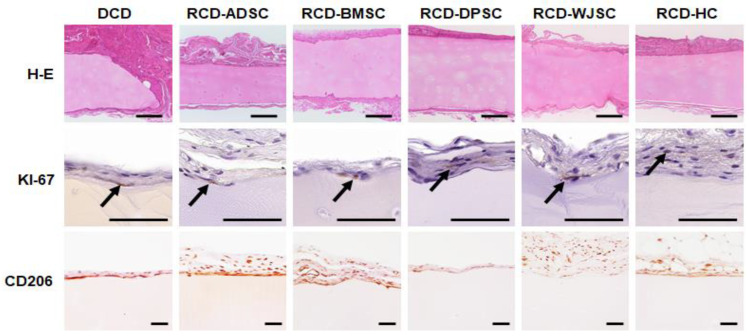
Histological analysis of samples grafted in vivo in laboratory animals, with H-E and immunohistochemistry for KI-67 and CD206. Arrows indicate representative cells with positive KI-67 staining. Scale bars: 200 µm.

After 60 days of in vivo follow-up, we found that all grafted samples were well integrated in the host animal, and no signs of necrosis, infection, hemorrhage, tumorigenesis or rejection were found in any of the animals. In histological terms, all grafts were surrounded by connective tissue with abundant collagen and host cells that tended to encapsulate the grafted biomaterial. No microscopic signs of rejection, infection, necrosis or other side effects were detected. In general, the grafted material remained stable after in vivo implantation, and no signs of host tissue or blood vessel migration inside the disks were detected (Figure 7).

When cell proliferation was analyzed in the different samples grafted in vivo (Figure 7), we observed that less than 1% of the cells found on the surface of DCD (corresponding to host cells surrounding the grafted tissue) and RCD-HC showed positive staining for KI-67. However, RCD containing human MSC showed more than 3% KI-67-positive cells (3.5% for RCD-ADSC and RCD-BMSC, 3% for RCD-DPSC, and 4.6% for RCD-WJSC). We then quantified pro-regenerative M2 macrophages to evaluate immune cell reactions driven by the implant in the host animal. As shown in Figure 7, most cells recruited at the graft site were CD206-positive pro-regenerative M2 macrophages (61.0% in DCD, 92.4% in RCD-ADSC, 89.5% in RCD-BMSC, 76.9% in RCD-DPSC, 93.9% in RCD-WJSC and 85.5% in RCD-HC). However, all samples were negative for the T lymphocyte markers CD4 and CD8, as expected (data not shown).

**Figure 8:**
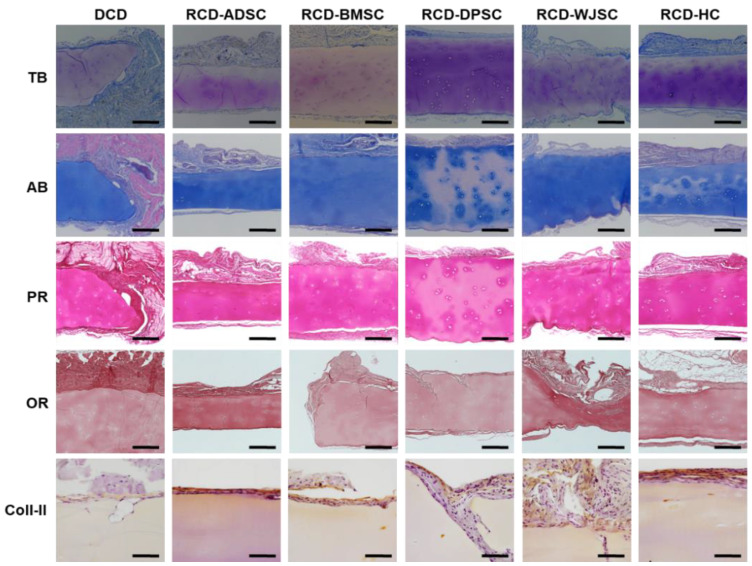
Histochemical and immunohistochemical analysis of extracellular matrix components in samples grafted in vivo in laboratory animals using TB, AB, PR and OR histochemistry and Coll-II immunohistochemistry. Scale bars: 200 µm.

To determine the effects of the in vivo environment on ECM composition in the grafted biomaterials, the disks were analyzed 60 days after implantation in laboratory animals (Figure 4 and Figure 8). The grafted materials were rich in proteoglycans and showed positive TB and AB staining. In general, the amount of proteoglycans in disks grafted in vivo was similar to the amount in non-grafted DCD, except for RCD-DPSC, which showed a significantly lower amount of AB-positive proteoglycans. The amount of proteoglycans in grafted disks was similar to the amount in S-CTR, except for grafted DCD and RCD-BMSC, which had smaller amounts of proteoglycans than the native control. Our analyses of collagen fibers with PR showed that the amount of collagen tended to be greater after in vivo implantation, and all samples grafted in vivo showed results similar to native S-CTR. Histochemical assays with OR disclosed the complete absence of elastic fibers in all samples grafted in vivo. Lastly, immunohistochemical assays for type II collagen showed significantly higher amounts in all sample types grafted in vivo compared to non-grafted DCD and S-CTR.

## 4. Discussion

Human cartilage typically has low regenerative potential, and novel replacement technologies able to improve cartilage regeneration are needed. Although numerous cell therapies and TE strategies have been proposed, the long-term results remain poor in most cases [38].

In the present work we used sturgeon cartilage as a biomaterial in TE. Although this biomaterial has been very recently used in cartilage TE, although using a different approach [21,22], its biological use is still preliminary and further research is in need. In general, this material offers several advantages compared to other types of scaffold. Our results suggest that its structure and function are analogous to human cartilage, and it is foreseeable that this biomaterial may mimic human cartilage more efficiently than other types of material. In addition, it is a natural material available in numerous fish farms, and the use of this product in TE could increase the commercial value of aquaculture products, as previously suggested for squid tissue used in TE [39]. Despite the potential usefulness of sturgeon tissues such as the skin [40] and the swim bladder [41,42] was previously proposed, very few works focused on the ex vivo and in vivo characterization of the decellularized sturgeon cartilage used in TE.

The sturgeon belongs to a primitive group of vertebrates in which the endoskeleton is formed by cartilage [43]. In general, the structure and molecular composition of this animal remain mostly unknown. However, the few published studies that compared human and sturgeon proteins suggest that homology is high for certain highly-conserved proteins such as Gla-rich protein [44] and insulin protein [45]. Because no data were available regarding the cartilage components, we first analyzed and compared human and sturgeon cartilage at three levels: in silico analysis of available sequences, UPLC, and histological analyses. Our results showed that the known collagen sequences in this fish were highly homologous to human collagen. Although whole sequences were not available for the sturgeon, and only partial protein sequences could be analyzed, these preliminary results are in agreement with previous results suggesting that fish and humans share approximately 70% of their genes [46]. When sturgeon and human cartilage were analyzed by UPLC, we found that both tissues consisted mainly of the major amino acids found in collagen fibers, and that the collagen proteins in both species contained the same amino acids. Finally, our preliminary analysis of the cartilage of both species with histological methods and techniques suggests that the histological structure and composition of sturgeon cartilage may be very similar to human cartilage, especially regarding the ECM. Although a comprehensive histological and histochemical characterization of sturgeon cartilage should still be carried out, the present results suggest that human and sturgeon cartilage may be very similar, and thus opens the door to future applications of this material.

Decellularized cartilage from different sources has been used previously in TE with positive results [47,48]. However, sturgeon cartilage has rarely been used for this purpose, and decellularization protocols specific for sturgeon cartilage have not been optimized and there is no general consensus available to date. In general, the use of xenografts obtained from other species requires previous decellularization in order to eliminate all non-human cells without significantly altering the ECM [28,37,49]. In the present work, we applied a combination of several previously described physical, chemical and enzymatic decellularization methods [27,28,29] to sturgeon cartilage, as recently described for different species of sturgeon [21,22]. As a highly dense, compact tissue, cartilage is considered to be difficult to decellularize, mostly because of the low penetrability of decellularization agents in the dense ECM, and thus their limited efficiency in removing all cells and cell debris [12]. For this reason, we used a combined decellularization protocol in which several types of highly effective detergents were used together with gentle enzymatic treatment and initial osmotic shock with distilled water. Although specific studies should be carried out to determine the most efficient decellularization method for this type of tissue, our results were promising and allowed us to obtain a suitably decellularized biomaterial without significantly altering the ECM structure and composition. This combined protocol allowed us to generate decellularized sturgeon cartilage disks for direct use in TE, which, to our knowledge, had not been described previously. Instead, the very recent reports related to sturgeon cartilage decellularization are based on a combined protocol that included tissue trimming and homogenization, solubilization and lyophilization to the desired three-dimensional shape [21,22]. In comparison to these reports, our method is simpler and more straightforward, and it is very likely that the structure and composition of the native cartilage remain better preserved in the final product.

In general, we found that cartilage proteoglycans as determined by TB and AB histochemistry were appropriately preserved in tissues decellularized with our method. Although both staining methods are able to detect cartilage ECM proteoglycans, it was previously demonstrated that TB and AB have higher affinity for specific types of components such as sulfated proteoglycans, acidic polysaccharides and glycosaminoglycans [50], and the combined use of both methods can provide more comprehensive information on the tissue components present in the ECM. In contrast, we found that mature collagen fibers in sturgeon cartilage were not resistant to the decellularization process, as shown by the reduced PR staining intensity. These results are in agreement with previous reports demonstrating that proteoglycans are highly resistant to detergent-based decellularization, whereas fibrillar ECM components may be more affected by the decellularization protocol [28].

Once decellularized, the novel biomaterials were recellularized to generate a cartilage-like bioartificial tissue with potential future clinical applications. Tissue recellularization can be done with orthotypical cells such as chondrocytes in the case of cartilage [21], or alternative heterotypical cells able to differentiate to the desired cell type [24,25]. In our case, we found that human chondrocytes grew efficiently on the surface of the biomaterial, where cells attached to DCD during at least 4 weeks of ex vivo development, and showed good biocompatibility and negative expression of apoptosis markers, although the cells were unable to penetrate the biomaterial. In this regard, it is well known that cell penetration into this type of dense biomaterials is difficult, and several authors proposed the possibility of dissolving the decellularized biomaterials in acid in order to obtain a hydrogel in which cell could be incorporated [21,22]. To determine the biosynthetic activity of cells cultured on the DCD, we analyzed the different ECM components in cartilage-like tissues generated with human chondrocytes and maintained ex vivo. We found no changes in the tissue content of proteoglycans, elastic fibers or type II collagen, but a significant increase in the amount of mature collagen fibers. These results suggest that human chondrocytes cultured on DCD may be metabolically active and able to synthetize ex vivo some of the major components of human cartilage, as previously reported for other biomaterials [47] and for rabbit chondrocytes seeded on decellularized scaffolds [21]. Regardless of the cell type, cultured cells were previously found to be able to modify the composition of the ECM by synthetizing different ECM components ex vivo [28,48].

In addition, we tested different types of MSC as alternative cell sources for recellularization on DCD. All MSC types we tested ex vivo were also able to attach to the biomaterial surface, but were unable to migrate into the structure. Interestingly, we found that the composition of RCD-ADSC, RCD-BMSC and RCD-DPSC remained stable ex vivo. However, analysis of the ECM in biomaterials recellularized with WJSC showed an increase in collagen fibers, and no differences were found compared to native human cartilage in any of the fibrillar or non-fibrillar ECM components analyzed here. This finding may be explained by the intrinsic nature of WJSC. These cells derive from a mucous connective tissue able to produce large amounts of ECM components in the umbilical cord [51], and their ECM biosynthetic potential has been demonstrated in culture [24,52]. In addition, it was reported that the biological components of Wharton jelly are similar to those of articular cartilage [53], and WJSC have previously been used to generate bioartificial cartilage by TE [24,54]. Interesting, previous reports demonstrated that sturgeon cartilage decellularized with a different protocol including ECM solubilization were able to support human ADSC adhesion [22], although other types of MSC were not tested.

A crucial step in the development of tissue substitutes for clinical use is in vivo evaluation in animal models. Our tissue-like cartilage substitutes containing human cells were grafted in immunodeficient nude mice to evaluate their in vivo behavior without triggering immunological rejection of the human cells. The results showed that all grafted tissues were safe for the host animal, with no side effects detected in any of the animals, and that the grafted cells were functional and able to proliferate in vivo. The fact that the animal model used here was immunodeficient does not allow us to establish definite conclusions regarding the biocompatibility of the grafted materials. However, earlier studies found that nude mice are severely immunodeficient for T lymphocytes [55], but other types of immune cells such as B lymphocytes and macrophages may be partly functional [56,57]. Our preliminary analyses demonstrated the complete absence of T cell function as determined by CD4 and CD8 immunohistochemistry, and the presence of abundant pro-regenerative M2 macrophages instead of pro-inflammatory M1 macrophages [58,59]. Although the relevance of these findings remains to be determined through further in-depth analyses in immunocompetent animal models, these initial results point to a positive effect of these tissues once implanted in vivo and are in agreement with previous works showing that decellularized sturgeon scaffolds are biocompatible after implantation in nude mice [21]. Future experiments should be carried out using additional immune cell markers such as CD68 and CD86.

We found that the bioartificial cartilage substitutes grafted in vivo remained highly stable for 60 days, with few signs of neovascularization or tissue remodeling by the host cells. These results may be explained by the highly dense structure of cartilage ECM [12], and suggest the possibility of using these tissue substitutes to replace damaged organs that must remain stable for long periods, as in the ear, nose and trachea, as well as other types of noncartilaginous tissues as in the human cornea. Future biomechanical analyses should determine the suitability of these tissue substitutes to withstand the forces associated to the natural environment. However, the grafted materials became encapsulated by host connective tissue which surrounded the grafts. This phenomenon is common in dense biomaterials that remain stable after long in vivo follow-up periods, whereas rapidly degradable materials tend to undergo an internal inflammatory response that breaks up the material into small fragments that are subsequently phagocytosed by host cells [60]. The fact that the tissue-like substitutes generated from sturgeon cartilage showed low biodegradability supports their future biological use to generate human tissues by TE, although functionalization protocols should be developed to increase the permeability of these materials and thus facilitate cell entry and colonization.

Strikingly, we also found that the in vivo environment was able to modify the grafted scaffold and remodel its ECM composition. In general, our tissue-like substitutes implanted in animals showed few variations in proteoglycan content, whereas collagen fibers were significantly increased, especially type II collagen. For proteoglycans and type II collagen, it is noteworthy that the lowest contents of these components tended to correspond to acellular DCD grafted in vivo, suggesting that cells cultured on the surface of the biomaterial may also play a role in the synthesis and maintenance of the main ECM components in vivo, and that the biomaterial used here could provide a chondroinductive environment to the cells cultured on its surface. In contrast, the highest concentration of type II collagen was found in RCD-HC, suggesting that the biosynthetic activity of human chondrocytes may be responsible for ECM remodeling not only ex vivo, but also after grafting in animals. Interestingly, all RCD recellularized with MSC showed positive type II collagen synthesis in vivo, although differences among cell types were detected. Type II collagen is one of the main components of human articular cartilage, and its presence and physiological role are crucial for appropriate functioning of this structure [61]. The fact that all four MSC analyzed here were able to induce the synthesis of mature collagen and type II collagen in vivo suggests that these cells may have the potential to differentiate to the chondrogenic cell lineage, as previously demonstrated [22,25,54]. In this connection, we also found that the highest in vivo biosynthetic activity among the four types of MSC was seen for ADSC, whereas the lowest activity was found for DPSC and BMSC. This is in agreement with previous results suggesting that ADSC can be efficiently differentiated to the chondrogenic lineage with decellularized porcine [47] and sturgeon [22] cartilage biomaterials.

The present study has several limitations. First, our biocompatibility and biodegradation results should be viewed with caution and taken as preliminary. In addition, new experiments should determine the structure and in vitro degradation rate of the novel biomaterial described here, and should investigate which properties favor cell migration into the biomaterial other than generating a hydrogel by cartilage solubilization as recently suggested [21,22]. In addition, both the DCD and RCD should be characterized using biomechanical testing to determine their biomechanical properties such as the Young modulus, elastic modulus or shear modulus, as previously described for the human native cartilage [62]. Finally, future studies should determine the potential clinical usefulness of the sturgeon cartilage biomaterial to generate human cartilage substitutes by TE, along with other types of bioartificial tissues such as human cornea. The stability of this biomaterial along with its intrinsic stiffness and transparency suggest that sturgeon cartilage may be potentially useful in cornea TE. In addition, the in vivo usefulness of the tissue substitutes generated in the present work, and the specific cartilage zone that could be treated with these substitutes, should be determined by orthotopic implantation at the articular surface of relevant animal models. In this regard, we may hypothesize that our tissue substitutes could be more suitable for replacement of the middle and deep zones of the articular cartilage, since these zones have been described to have lower cell density and higher amount of ECM components than the superficial zone [63].

In summary, our preliminary findings suggest that biomaterials obtained from decellularized sturgeon cartilage may be suitable for the generation of tissue substitutes with different cell sources. Future studies should be carried out to determine the clinical usefulness of this approach, and to improve the applicability of these biomaterials by modifying their biological and biomechanical properties.

## 5. Patents

O.O.-A., R.C., O-D.G.-G., V.C. and M.A. are coauthors of patent application number P202031030, “Sturgeon Cartilage Bio-material For Tissue Regeneration”.

## Data Availability

The data presented in this study are available on request from the corresponding authors.

## Supplementary Materials

The following are available online at https://www.mdpi.com/article/10.3390/biomedicines9070775/s1, Supplementary Figure S1. Alignment analysis of the previously known human and sturgeon collagen sequences using the MultAlin program.
