# Supplementary material for: Generation and Evaluation of Novel Biomaterials Based on Decellularized Sturgeon Cartilage for Use in Tissue Engineering
Source: Biomedicines. 2021 Jul 4;9(7):775. doi: 10.3390/biomedicines9070775 (PMC8301329; doi:10.3390/biomedicines9070775)

Supplementary Figure S1. Alignment analysis of the previously known human and sturgeon collagen sequences using the MultAlin program. A: alpha-1 chain of collagen type I; B: alpha-2 chain of collagen type I; C: alpha-1 chain of collagen type II. For each comparison, the human and sturgeon sequences are shown along with the consensus sequence obtained between both sequences. High consensus is shown in red and low consensus is shown in blue for each amino acid.

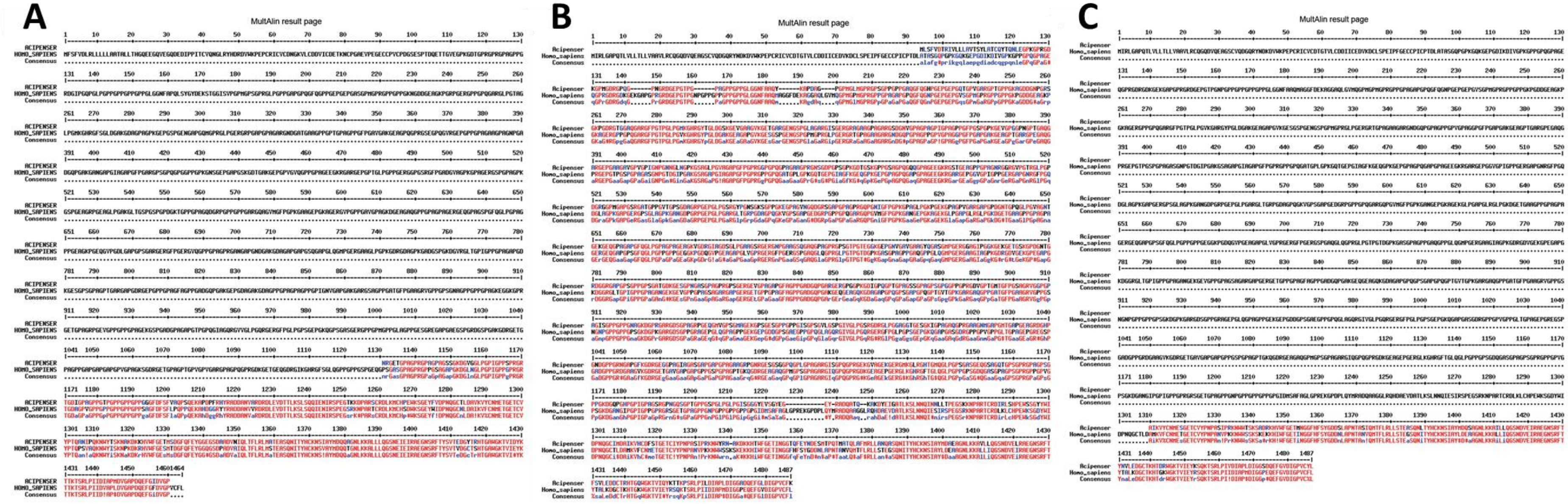

Supplement: Supplementary file 1 [file biomedicines-09-00775-s001.zip › biomedicines-1273219-supplementary.pdf]
